# Comparative essentialome analysis of six Pectobacteriaceae strains using the TNSEEK pipeline identifies conserved and strain-specific fitness determinants

**DOI:** 10.1099/mgen.0.001762

**Published:** 2026-09-11

**Authors:** Julie Baltenneck, Loïc Couderc, Jacques Pédron, Guillemette Marot, Areski Flissi, Hélène Touzet, Erwan Gueguen, Marie-Anne Barny, Guy Condemine

**Affiliations:** 1 Univ Lyon, Université Lyon 1, INSA de Lyon, CNRS UMR 5240 Microbiologie Adaptation et Pathogénie, Villeurbanne, France; 2 Univ. Lille, CNRS, Inserm, CHU Lille, Institut Pasteur de Lille, US 41 - UAR 2014 - PLBS, Lille, France; 3 Sorbonne Université, INRAE, Institut d'Écologie et des Sciences de l'Environnement de Paris, Paris, France; 4 Univ. Lille, CHU Lille, Inria, ULR2694 – Metrics - Datavers, Lille, France; 5 Univ. Lille, CNRS, Centrale Lille, UMR 9189 - CRIStAL - Centre de Recherche en Informatique Signal et Automatique de Lille, Lille, France

**Keywords:** *Dickeya*, essentialome, *Pectobacteriaceae*, *Pectobacterium*, transposon sequencing (Tn-seq)

## Abstract

Transposon sequencing (Tn-seq) is a powerful technique for defining the essential genes required for bacterial survival. However, gene essentiality can vary significantly across taxonomic levels, and comparing large Tn-seq datasets from multiple strains presents considerable analytical challenges. To address this, we developed TNSEEK, a fully automated bioinformatics pipeline for the systematic and comparative analysis of Tn-seq experiments. We applied TNSEEK to analyse newly generated data for six soft rot *Pectobacteriaceae* strains, encompassing species from the *Dickeya* and *Pectobacterium* genera, grown in a rich medium. This approach identified a core essentialome of 225 genes, primarily involved in fundamental cellular maintenance, conserved across all 6 strains, a set comparable in size to that of the neighbouring *Enterobacteriaceae* family. Only a few genus-specific essential genes were found, highlighting interesting distinct metabolic capabilities between *Dickeya* and *Pectobacterium* genera. In striking contrast, we discovered a large variable essentialome comprising 181 strain-specific genes, many of which are of unknown function. A portion of these strain-specific essential genes are components of defence systems and prophage genomic regions. The unexpected essentiality of selected components of these modules is consistent with cellular dependency on cognate toxic, restriction or immunity functions encoded by defence-associated loci under the tested growth condition. Furthermore, a comparison with the *Escherichia coli* essentialome demonstrates that discrepancies in gene essentiality can often be attributed to differences in growth conditions, particularly temperature, as well as variations in genetic redundancy. In conclusion, the TNSEEK pipeline provides a reproducible framework for comparative analysis of mariner/Himar1 Tn-seq datasets across multiple strains.

**Impact Statement**

*Dickeya* and *Pectobacterium* are two genera of the *Pectobacteriaceae* family that contain mainly plant pathogenic bacteria. To analyse the diversity within bacteria of this family, we performed a transposon sequencing (Tn-seq) analysis on six strains representing a range of ecological niches. To this aim, we developed a bioinformatics pipeline, termed TNSEEK, which allows the comparative analysis of results across diverse experimental conditions and multiple strains. Applied to growth in rich medium of the tested strains, it allowed the identification of an essentialome of 225 genes at the family level, a few genus-specific essential genes but a large essentialome comprising 181 strain-specific genes, many of which are of unknown function. Thus, TNSEEK proved its ability to analyse a large dataset coming from different Tn-seq experiments, and it provides a flexible framework for handling multiple strains and experimental conditions.

## Data Summary

The sequence of *Pectobacterium versatile* A73-S18-O15 and *Dickeya parazeae* A586-S18-A17 have been deposited at the National Center for Biotechnology Information (NCBI) platform under assembly accession numbers GCF_020865565.1 and GCF_020520245.1, respectively. The Tn-seq datasets newly generated for the six soft rot *Pectobacteriaceae* strains have been deposited under BioProject PRJNA1389912, with SRA accession numbers indicated in Table S4.

## Introduction

In 2005, Samson *et al*. revised the genus *Pectobacterium*, which belongs to the *Enterobacterales* order and established the distinct genus *Dickeya* [1]. *Pectobacterium* and *Dickeya* belong to the *Pectobacteriaceae* family, within the order *Enterobacterales* and the class *Gammaproteobacteria*. *Pectobacteriaceae* is phylogenetically close to the *Enterobacteriaceae* family within the *Enterobacterales* order. *Pectobacterium* and *Dickeya* genera encompass phytopathogenic bacteria responsible for soft rot symptoms. Subsequently, epidemiological and taxonomic investigations have led to the identification of numerous novel species within both genera. Currently, 23 recognized species of *Pectobacterium* and 13 species of *Dickeya* have been characterized [2, 3]. Soft rot *Pectobacteriaceae* (SRP) exhibit a broad host range, encompassing both monocotyledonous and dicotyledonous plant species. The majority of these bacteria have been isolated from soft rot symptoms in a diverse array of infected plants. These plants frequently include species of significant agronomic importance, such as potato, rice, maize, cabbage and ornamentals. These bacteria are capable of inducing disease during both pre-harvest production and post-harvest storage. Given the frequent monitoring of these crops during growth and storage, the isolation of plant-infecting strains is regularly achieved. However, certain SRP strains can also be found in alternative environments such as weeds or water [4], and some species have been mostly isolated from freshwater sources and have not been associated with plant hosts. Examples include *Dickeya aquatica*, *Dickeya lacustris*, *Dickeya undicola* and *Pectobacterium aquaticum* [5]. Whole-genome sequencing reveals genome size variability both intragenerically and intraspecifically. Specifically, within the *Dickeya* genus, average genome sizes range from 4.02 Mb for *Dickeya poaceiphila* to 5.35 Mb for *Dickeya dadantii*. Furthermore, intraspecific variation in genome size is evident, with *Dickeya zeae* exhibiting a range from 4.56 to 4.93 Mb and *D. dadantii* ranging from 4.66 to 5.35 Mb [3].

Genomics also allows the development of high-throughput technologies, such as transposon sequencing (Tn-seq), that have facilitated the identification of numerous essential genes within a given bacterial strain [6]. However, interpretations based on the analysis of a single, typically laboratory-adapted strain should be approached with caution. To mitigate this limitation, Tn-seq analysis of multiple strains is imperative. For instance, Ghomi *et al*. [7] analysed 14 strains to define a core set of 201 essential genes for the *Enterobacteriaceae* family. Their analysis of individual strains revealed between 300 and 440 essential genes in each respective strain. Similarly, an investigation involving 17 strains of *Streptococcus pneumoniae* identified 206 core essential genes, 186 genes present in all strains but essential in only a subset and 128 essential genes located within the accessory genome [8]. These findings underscore the strain-dependent nature of gene essentiality and emphasize the necessity of analysing and comparing data from a substantial number of strains to achieve a robust understanding of essentiality at the species, genus and family levels.

To enable such a comparative analysis of extensive Tn-seq datasets generated from multiple bacterial strains, we developed a fully automated bioinformatics pipeline, termed TNSEEK. This pipeline encompasses the entire data processing workflow, from raw reads to the prediction of essential genes, and permits the comparative analysis of results across diverse experimental conditions and multiple strains. TNSEEK was utilized to analyse Tn-seq data obtained from six SRP strains, comprising three *Dickeya* species (*D. dadantii*, *Dickeya parazeae* and *Dickeya fangzhongdai*) and three *Pectobacterium* species (*Pectobacterium atrosepticum*, *Pectobacterium versatile* and *P. aquaticum*), representing a range of ecological niches. The application of the TNSEEK pipeline facilitated the determination of the core essential genome of SRP and allowed for the differentiation of functions associated with core and strain-specific essential genes.

## Methods

### Bacterial strains and growth conditions

All bacterial strains and oligonucleotides employed in this study are detailed in Tables S1 and S2 (available in the online Supplementary Material). *Escherichia coli* cells were cultured at 37 °C in Luria–Bertani (LB) medium. *Dickeya* and *Pectobacterium* cells were cultured at 25 °C in 50% tryptic soy broth (TSB; 20 g l^−1^) medium (Formedium Ltd, Norfolk, UK). When required, media were solidified by the addition of 15 g l^−1^ agar. Antibiotics were supplemented at the following concentrations: 100 µg ml^−1^ for ampicillin (Amp), 25 µg ml^−1^ for kanamycin (Km) and 25 µg ml^−1^ for chloramphenicol (Cm).

### Construction of the transposon library

For each bacterial strain, 10 OD units at 600 nm (OD_600_.U) of an overnight culture were combined with 10 OD_600_.U of an overnight culture of *E. coli* MFDpir/pSamEC. The pSamEC vector is a suicide mobilizable plasmid containing a kanamycin resistance gene (Km^R^) flanked by mariner inverted repeat sequences with an *MmeI* restriction site and the *himar1*-C9 transposase gene under the control of the *lac* promoter [9]. This transposon specifically inserts at TA dinucleotide sites within the genome. This mixture was centrifuged at 5,000 r.p.m. for 10 min at ambient temperature. The resulting bacterial pellet was resuspended in 100 µl of TSB 50% and dropped onto a TSB 50% agar plate supplemented with 57 mg l^−1^ diaminopimelate. Following incubation overnight at 30 °C, bacteria were collected and resuspended in 1 ml of TSB 50%. A sample was then diluted and plated onto a TSB 50% agar plate containing kanamycin to assess mutagenesis efficiency. The remaining bacterial suspension was spread onto TSB 50% agar plates with kanamycin and incubated for 48 h at 30 °C. Bacteria were harvested from the plates by adding TSB 50%. The cultures were adjusted to a 60 OD_600nm_.U, mixed with an equal volume of 80% glycerol, aliquoted and stored at −80 °C.

### DNA preparation for Tn-seq high-throughput sequencing

Genomic DNA was extracted from ~90 mg of bacterial cells for the control strain libraries and from cultures grown for at least seven generations in 50% TSB at 25 °C for the mutant libraries using the Wizard Genomic DNA Purification kit (Promega), following the manufacturer’s protocol. Subsequent steps followed precisely the methodology described by Baffert *et al*. [10].

### Sequencing and genome assembly

High-quality, complete genome sequences were generated using a hybrid sequencing approach, combining Oxford Nanopore Technologies (ONT) long-read and Illumina short-read sequencing. *P. versatile* strain A73-S18-O15 and *D. parazeae* strain A586-S18-A17 were grown in 2 ml of liquid LB medium at 28 °C. The bacterial cells were then harvested by centrifugation and genomic DNA was extracted. Illumina sequencing was performed at the Next Generation Sequencing Core Facility of the Institute for Integrative Biology of the Cell (I2BC). Nextera DNA libraries were prepared from 50 ng of high-quality genomic DNA. Paired-end sequencing (2×75 bp) was conducted on an Illumina NextSeq 500/550 instrument using a High Output 150-cycle kit, resulting in ~60-fold coverage for strain A586-S18-A17 and 180-fold coverage for strain A73-S18-O15. Long-read sequencing was conducted at the Genotoul platform (https://www.genotoul.fr), employing Oxford Nanopore GridION technology over a 72 h run. This yielded 447,101 reads for strain A586-S18-A17 and 335,205 reads for strain A73-S18-O15, with average read lengths of 4,473 and 5,173 bp, respectively. The resulting sequencing depths were 417-fold for A586-S18-A17 and 361-fold for A73-S18-O15. Hybrid genome assembly, utilizing reads from both long-read and short-read sequencing strategies, was performed using Unicycler v0.4.9 with default parameters [11]. The assembled genomes were deposited and annotated on the National Center for Biotechnology Information (NCBI) platform, and accession numbers for each genome are indicated in Table S1 (https://www.ncbi.nlm.nih.gov/assembly).

An orthologous gene matrix was computed for the six analysed genomes using the Orthologous Matrix (OMA) standalone package (https://omabrowser.org/standalone/) [12]. Functional annotation of essential genes was performed using eggNOG-mapper v2 [13] against the eggNOG v5.0 database [14]. The resulting OMA orthogroups and functional annotations are presented in Table S3.

### Description of TNSEEK

To enhance the analysis of a substantial volume of sequencing experiments, we have developed an entirely automated bioinformatics pipeline, TNSEEK, which encompasses the entire processing chain – from raw reads to the prediction of essential and conditionally essential genes. TNSEEK relies primarily on statistical approaches provided by the TRANSIT software suite [15] and offers additional features to facilitate comparison of results across multiple conditions and strains. TNSEEK can only be applied to compatible mariner/Himar1 TA-site-based datasets and provides a reproducible framework for handling multiple strains and experimental conditions.

**Fig. 1.**
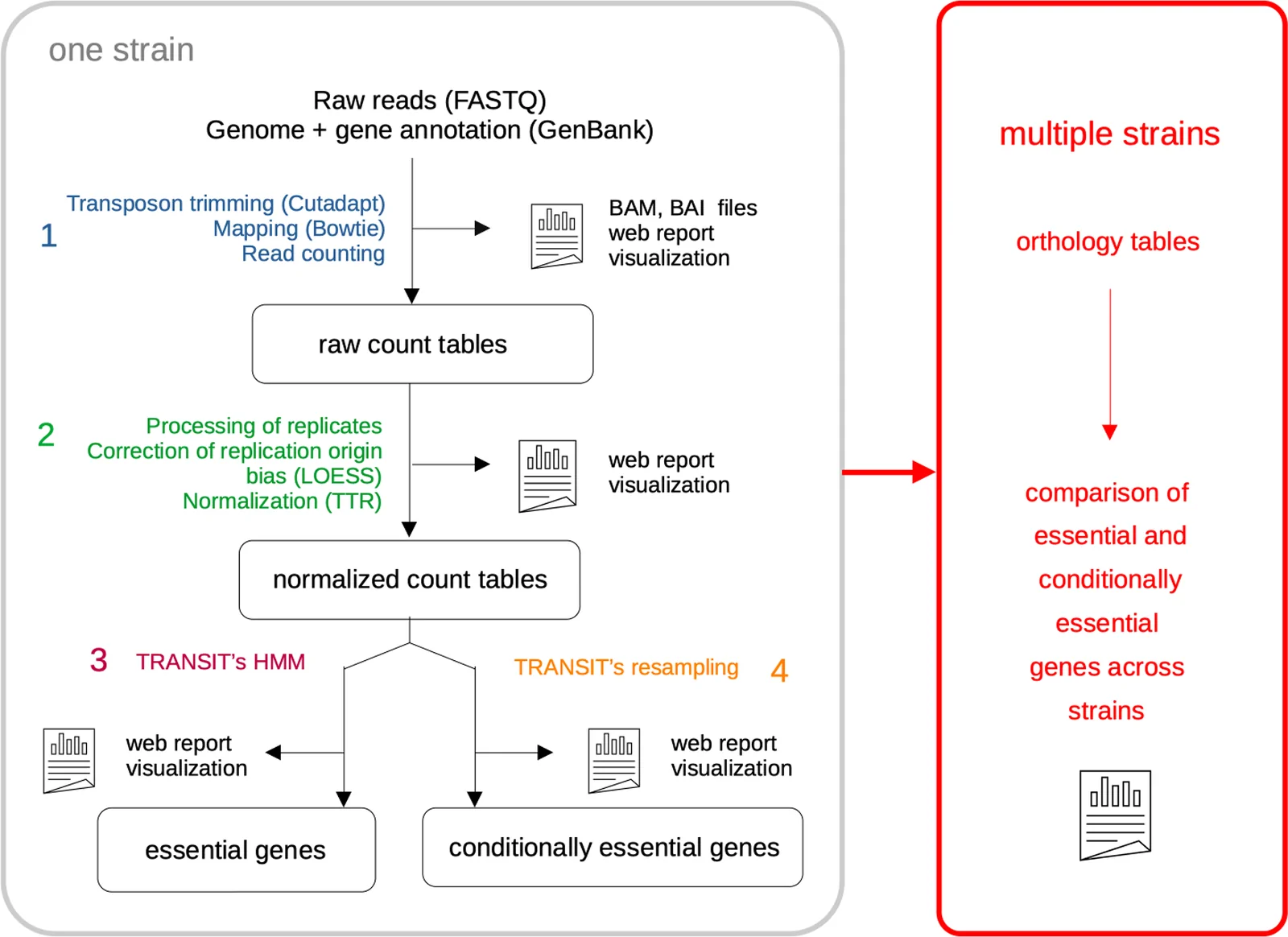
Overview of the TNSEEK pipeline. Illustration of the main steps of the pipeline, beginning with the raw sequencing reads and culminating in the identification of essential and conditionally essential genes. The pipeline initially processes data from each strain independently, followed by a comparative analysis of results across strains at the orthology family level.

An overview is provided in Fig. 1, and its key steps are outlined below:

Step 1: Read mapping and construction of count tables. TNSEEK was run on demultiplexed FASTQ files generated from mariner/Himar1 Tn-seq libraries. In the pSam_Ec protocol used here, the sequencing platform demultiplexed the samples but retained the 5-nt internal barcode at the 5′ end of each read. The reads processed by TNSEEK therefore had the structure 5′-[5-nt barcode]-[16–17 nt genomic DNA tag]-[transposon-derived sequence]-3′. TNSEEK was run with Cut=5 to remove the internal barcode. The user-defined transposon-derived sequence ACAGGTTGGATGATAAGTCCCCGGTCTT was then searched downstream of the genomic tag and trimmed using Cutadapt [16]. The minimum overlap between the read and the transposon sequence was set to 7 nt, and a maximum error rate of 0.05 was allowed. After trimming, only genomic tags of 16 or 17 nt were retained and mapped to the reference genome with Bowtie2 using end-to-end alignment [17]. To build the raw count tables, only tags mapping uniquely to the genome and whose 3′ end mapped adjacent to a TA dinucleotide were retained; all other reads were discarded. This TA-site filtering is consistent with the insertion specificity of the mariner/Himar1 transposon. In the current implementation, TNSEEK is designed for mariner/Himar1-based Tn-seq datasets in which insertions occur at TA dinucleotides, including some already publicly available [18–20]. The transposon sequence and trimming parameters are user-configurable; the default sequence in the public interface is ACAGGYTGGATGATAAGTCCCCGGTCT, where Y is the IUPAC ambiguity code for C or T. However, datasets generated with Tn5 or other transposons not restricted to TA dinucleotides would require modification of the read-processing and insertion-site definition steps before analysis with TNSEEK/TRANSIT.

Step 2: Normalization and quality control. We began by applying locally estimated scatterplot smoothing normalization (local weighted regression) to correct for replication origin bias. We then eliminated non-biological variability between tables using trimmed total reads (TTR) normalization. In the current implementation, TNSEEK applies TTR normalization and does not allow users to select alternative TRANSIT normalization methods directly from the graphical interface. TTR was selected because it is recommended in TRANSIT for comparative Tn-seq analyses and compensates for differences in library saturation. Other methods, such as totreads and nzmeans, were evaluated but were less robust for the datasets analysed here. Since TNSEEK is modular, users can nevertheless generate Wiggle files outside the pipeline and then proceed with the downstream analysis modules. This step resulted in one normalized count table per strain and condition, along with several quality control metrics, taken from TRANSIT: sum, mean, skewness and kurtosis of the read counts in the dataset, the density (i.e. the fraction of sites with insertions), as well as quantile–quantile and quality control plots. The pipeline also performed pairwise comparisons of replicates: For each strain and each condition, we computed the Pearson correlation coefficient between TA sites for each pair of replicates.

Step 3: Detailed characterization of essential genes. Based on the read counting, genes were classified into four categories: essential (ES), growth defect (GD), growth advantage and non-essential (NE). For that, we used the hidden Markov model approach introduced in [15] that models the genome as a sequence of four hidden states calculating the likelihood of observed insertion counts for each gene.

Step 4: Characterization of conditionally essential genes: TNSEEK can also identify conditionally essential genes. TRANSIT’s RESAMPLING method is used to compare experimental conditions with the control conditions. TNSEEK features an intuitive user interface and facilitates the interpretation of results by generating a comprehensive report accessible via any standard web browser. This report enables users to systematically navigate through the analysis process, accompanied by a diverse array of figures and graphical representations. For a holistic understanding, all findings are synthesized into global tables at the strain level. This step is not used in the present study.

Step 5: Tn-seq result comparative analysis. TNSEEK provides additional insights to compare essential and conditionally essential genes across species, utilizing OMA analysis orthogroup [12] links between strains to enhance comparative analysis. In this study, the comparative module was run using OMA-derived orthogroups. TNSEEK currently expects an orthogroup table formatted according to the OMA output structure; however, orthology assignments generated by other clustering tools could in principle be used if converted into the expected TNSEEK input format, linking each gene or locus tag to an orthogroup identifier across strains. This allows to differentiate between a TNSEEK-predicted conserved, core essential genes shared between all strains and variable combinations of strain-specific essential genes, which is critical for understanding the diversity and adaptability of bacterial species.

**Fig. 2.**
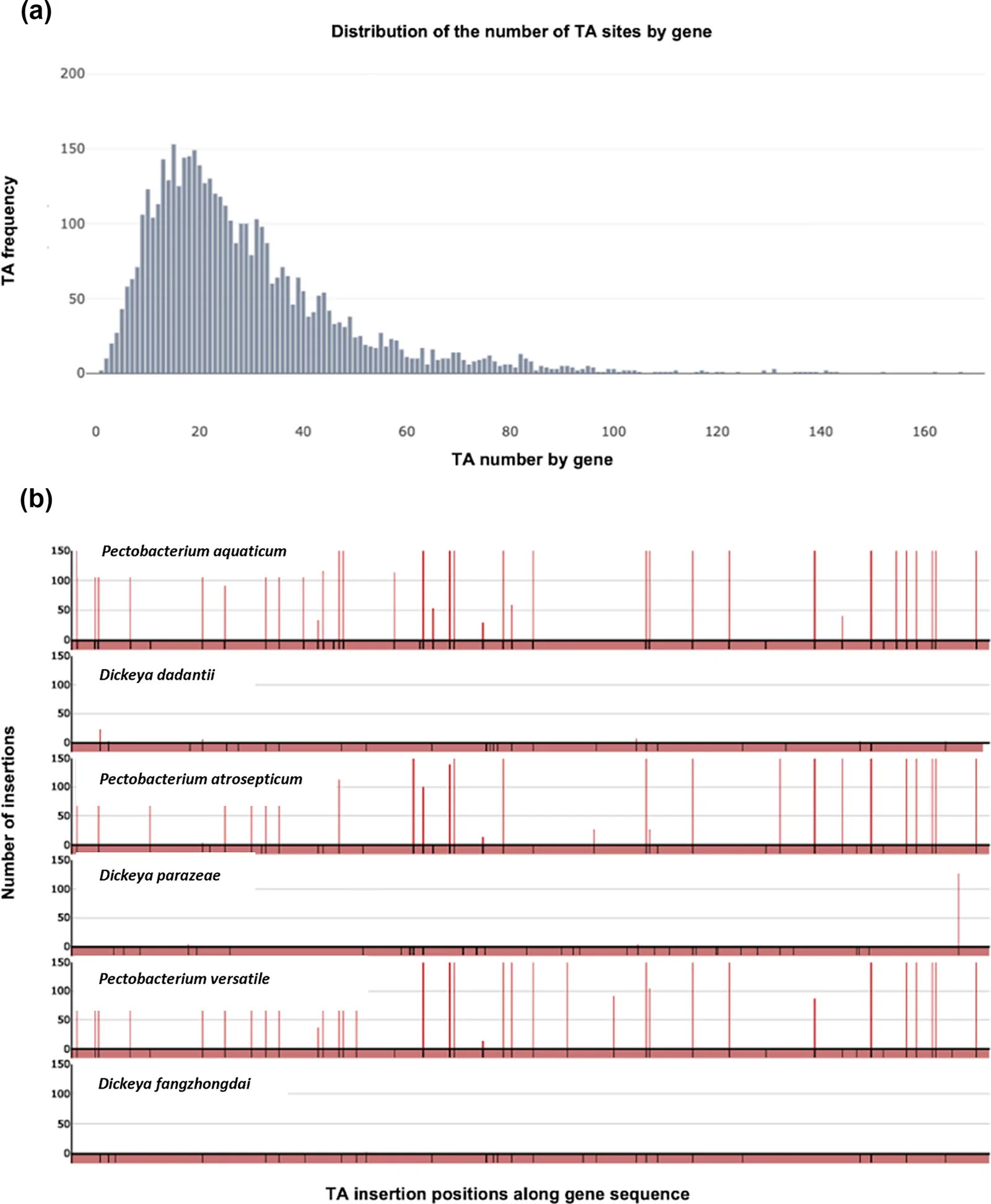
Examples of graphical outputs generated by the visualization tool of the TNSEEK pipeline. (a) Distribution of the number of TA sites per gene in *D. dadantii* 3937. (b) Insertion visualization for an essential genus-specific gene (orthogroup OMA03793). The figure clearly shows numerous insertions for the *Pectobacterium* genus (NE gene) and an absence of insertion for the *Dickeya* genus (essential gene). Black lines represent TA insertion sites, and red lines indicate the number of insertions along the gene.

Step 6: TNSEEK visualization. TNSEEK also incorporates a visualization tool for the detailed examination of individual genes and comparison between orthologous genes (Fig. 2).

TNSEEK comes with a user-friendly interface and facilitates result interpretation by generating a comprehensive report that can be conveniently accessed through any web browser. This report enables users to navigate through the analysis process step by step, accompanied by a diverse range of figures and graphs. For a holistic understanding, all results are summarized in global tables at the strain level, providing a comprehensive overview. Additionally, TNSEEK offers additional insights into essential genes and conditionally essential genes across species with orthology links between strains, thus facilitating comparative analysis. Lastly, TNSEEK includes a visualization tool dedicated to investigating individual genes. The computational core of TNSEEK is implemented in Python and R. The graphical user interface, specifically the web application designed for data exploration and visualization, is developed using R Shiny and SQLite. The report generation is facilitated by R Markdown. The complete pipeline is readily installable and executable as a Docker image. Source code and detailed documentation are available at https://gitlab.cristal.univ-lille.fr/bonsai/tnseek.

### Analysis of essential genes

**Fig. 3.**
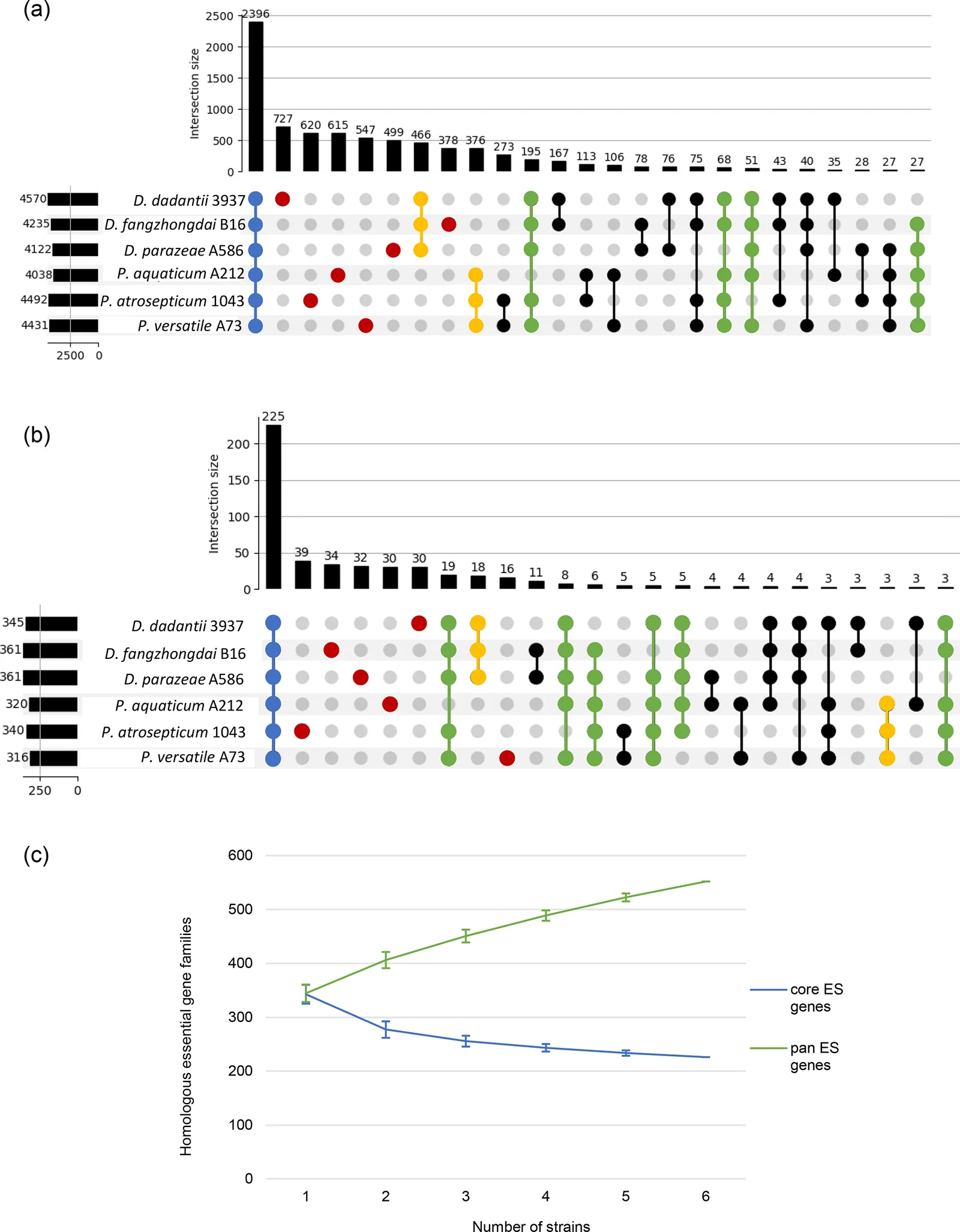
Shared genes and shared ES genes between the six studied strains. (a) Shared genes between the strains. (b) Shared ES genes between the strains. Blue, core genes shared by the six strains; red, strain-specific genes; yellow, genus-specific genes; green, genes shared by five strains out of six; black, other combinations. The number of genes is indicated on the top of each bar. The graph depicted on the left represents the total number of genes (**a**) or ES genes (**b**) per strain. Only the combinations with a gene number of more than 25 are shown for (**a**) and of more than two are shown for (**b**). (c) Rarefaction and accumulation analysis of ES genes. The graph shows the estimated sizes of the core and pan essential genes according to the number of strains added (up to six). Randomized genome sampling was performed 100 times. sd are shown as error bars.

To validate the essential gene predictions generated by the TNSEEK pipeline, several confirmatory criteria were established. Firstly, gene duplications within each strain’s genome were identified by conducting all-against-all nucleotide sequence similarity searches using blast 2.13.0+ with an *E*-value threshold of 10^−5^. Genes with duplicated copies were flagged as ES DU standing for ‘duplicated’ (Table S3) and excluded from the high-confidence essential gene set. In parallel, during read processing, tag mapping to multiple genomic positions was discarded before construction of the count tables. This conservative strategy reduces false-positive essentiality calls caused by ambiguous mapping in duplicated regions, although it may also remove genuine cases in which only one copy of a duplicated gene is functional or required under the tested condition. Secondly, given the potential polar effects of mariner transposon insertions, the operon structure of TNSEEK-predicted essential genes was analysed as described in the Results section. UpSet plots were generated using the Python implementation of the UpSet library (Fig. 3a, b) [21]. Intact phage regions within the six genomes were identified by PHASTEST (https://phastest.ca/). Rarefaction and accumulation analysis were performed with the presence/absence matrix of homologous gene families using the script rarefaction_curve.R and accumulation_curve.R under R version 4.0.2 [22].

## Results

### Genomic diversity and core genome of the six selected *Pectobacteriaceae* strains

In this study, we compared the essential genes of six SRP strains, selected to represent a variety of ecological niches. The chosen species include three from the *Dickeya* genus (*D. dadantii*, *D. parazeae* and *D. fangzhongdai*) and three from the *Pectobacterium* genus (*P. atrosepticum*, *P. versatile* and *P. aquaticum*). This selection encompasses a broad ecological range: *P. versatile* is isolated from both plants and surface water [2, 23], whereas *P. atrosepticum* is mainly restricted to *Solanaceae* [2], and *P. aquaticum* was found exclusively in waterways [23, 24]. Similarly, within the *Dickeya* genus, *D. fangzhongdai* occupies diverse habitats, including plants and aquatic environments [25–27], *D. parazeae* is frequently found in water [23, 28] and infects herbaceous plants like maize and rice and *D. dadantii* is primarily associated with dicotyledonous plants [3].

Two of these strains, *P. atrosepticum* SCRI1043 and *D. dadantii* 3937, are well-established model organisms whose genomes were among the first sequenced for their respective genera [29, 30]. The complete genomes of *P. aquaticum* A212-S19-A16 and *D. fangzhongdai* B16 have also been previously published [24, 27]. For this study, we sequenced and assembled the complete genomes of the remaining two strains: *P. versatile* A73-S18-O15 and *D. parazeae* A586-S18-A17.

A single replicon was observed for all strains analysed and no plasmid was detected. The genome sizes range from 4.46 Mb (*P. aquaticum*) to 5.06 Mb (*P. versatile*). The number of coding sequences varies from 4,038 (*P. aquaticum*) to 4,570 (*D. dadantii*). The number of pseudogenes ranges from 33 in *D. fangzhongdai* to 128 in *P. aquaticum*, while the number of CRISPR arrays varies from 1 (*P. aquaticum*) to 5 (*D. fangzhongdai*).

While the 6 strains share a core genome of 2,396 genes, a substantial portion of each genome is unique (Fig. 3a). The number of strain-specific genes ranges from 378 in *D. fangzhongdai* to 727 in *D. dadantii*, underscoring a high degree of genomic individuality. The analysis also reinforces the genomic divergence between the 2 genera, identifying 466 genes exclusive to the *Dickeya* strains and 376 genes exclusive to the *Pectobacterium* strains. As a rule, the number of genes shared exclusively in pairs between two species was higher for strains belonging to the same genus (from 273 to 76) than between species belonging to different genera (from 35 to 11). This genomic variability prompted us to investigate its functional consequences by defining and comparing the essential gene set for each strain using TNSEEK.

### Generation and validation of mariner-based transposon libraries

For Tn-seq, a Himar1 mariner transposon derivative was utilized. A mutant pool of ~1,000,000 colonies was generated for each of the 6 SRP strains by introducing the plasposon pSam_Ec [9] via conjugation from *E. coli*. DNA libraries were prepared in two technical replicates from this pool and analysed using high-throughput sequencing with the TNSEEK pipeline. The sequencing yielded high-quality data, with initial read counts ranging from 9.1 to 14.7 million per sample and final mapped reads between 8.6 and 13.8 million after filtering (Table S4). Mapping efficiencies were consistently high, between 84.42 and 96.08%, and the mean read depth per insertion site was uniform across samples (122.2–129.4 reads), providing sufficient coverage for statistical analysis. High Pearson correlation coefficients (0.96–0.99) between replicates confirmed the experimental reproducibility. The number of unique transposon insertion sites varied between the genera, with *Pectobacterium* strains showing higher counts (199,301–232,639) than *Dickeya* strains (166,816–194,008), reflecting differences in genome size and saturation. The TA insertion density, ranging from 37.9 to 62.7%, was sufficient for a comprehensive determination of gene essentiality (Table S4). All the data are available under BioProject number PRJNA1389912. The use of a standardized growth condition (TSB 50%) allowed for the direct comparison of essential gene repertoires, which facilitates the identification of both conserved core essential genes and species-specific fitness determinants among these related phytopathogenic bacteria.

### Defining the core and variable essentialome of *Pectobacteriaceae*

The initial step in our functional analysis involved processing the raw Tn-seq data with the TNSEEK pipeline to identify essential genes in each of the six strains. This first-pass analysis yielded a set of 665 orthogroups containing at least one potentially essential gene (Table S3). However, a known challenge with transposon-based methods like Tn-seq is the risk of generating false positives due to polar effects [31]. An insertion of the mariner transposon within a gene can not only disrupt that gene but also prevent the transcription of downstream genes located in the same operon. This can make these downstream genes appear essential, when in reality their function was never directly tested by an insertion.

To address this challenge and ensure the high quality of our dataset, we implemented a stringent and systematic curation strategy. The logic of our approach was to specifically identify and remove cases where essentiality is questionable due to a polar effect. We leveraged both known operon structures for *D. dadantii* 3937 [32] and predicted operon structures based on synteny for the other five strains. For each putative operon containing multiple genes identified as essential, we applied a conservative rule: only the gene located most distal from the promoter was retained as essential. The rationale is that an insertion in this last gene is the least likely to have a polar effect on any other gene, making its essentiality call the most reliable. However, this rule was applied with a critical exception to avoid inadvertently discarding truly essential genes. If a gene located upstream in an operon had a known essential orthologue in the well-characterized model organism *E. coli* K-12, it was maintained in our essential gene set [33]. This rule was used as a conservative curation criterion rather than as evidence that essentiality is necessarily conserved between *E. coli* and *Pectobacteriaceae*. We acknowledged that this operon-based filtering prioritizes specificity over sensitivity and may remove some truly essential upstream genes, while retaining upstream genes whose essentiality is supported by independent evidence from *E. coli*. This bioinformatic cross-validation allowed us to anchor our predictions in established biological knowledge. This meticulous approach enabled us to identify 455 orthogroups whose essentiality in at least 1 of the 6 analysed strains could not be attributed to polarity effects. Among the 210 orthogroups categorized as ES by the TNSEEK pipeline that could be prone to artefactual categorization due to the polarity of the Mariner transposon, 97 were categorized as essential in *E. coli* K12 by Goodall *et al*. [33]. We opted to include these 97 orthogroups in our subsequent analysis. The majority of these, 76 orthogroups, were categorized as ES across all 6 analysed strains. Through this rigorous, multi-step curation process, we successfully filtered out ambiguous cases. This refined our initial list to a final, high-confidence dataset of 552 orthogroups containing essential genes, with at least 1 ES gene across the 6 analysed strains (Table S5). This curated dataset formed the robust foundation for the subsequent comparative analysis of the core and variable essentialomes.

### The comparative analysis of the six strains discriminates between core, genus-specific and strain-specific essentialomes

Our comparative analysis, based on the high-confidence set of 552 essential orthogroups, highlights a distinction in the genetic requirements for survival among the SRP strains. We first identified a conserved core essentialome comprising 225 orthogroups (Fig. 3b, Table S6). These genes are required for growth in all six strains under the tested conditions. Rarefaction analysis indicated that this core essentialome reaches a plateau with the six strains analysed (Fig. 3c), suggesting that the dataset mostly captures the conserved essentialome under the tested condition. The total number of essential genes per strain was also relatively consistent, varying from 316 for *P. versatile*, 320 for *P. aquaticum*, 345 for *D. dadantii*, 340 for *P. atrosepticum* and 361 for *D. fangzhongdai* or *D. parazeae*.

In addition to this core set, we identified a large strain-specific essentialome, consisting of 181 genes pointing toward a considerable degree of functional individuality. The contribution of this variable set to each strain’s essentialome differed, ranging from 16 unique essential genes in *P. versatile* to 39 in *P. atrosepticum* (Fig. 3b, Tables S7–S12). This suggests that each strain may have developed specific dependencies on certain accessory genes. Only a few orthogroups exhibited genus-specific essentiality: 18 orthogroups were found to be exclusively essential for the three *Dickeya* species (Table S13). Even fewer, three orthogroups, were exclusively essential for the three *Pectobacterium* species (Table S14, Fig. 3b).

We then investigated the genetic basis for this strain-specific essentiality. In a high proportion of cases (87.6%), a gene was essential in a single strain because its orthologues were absent from the genomes of the other five strains (Table 1). This observation links the functional essentiality to the genomic diversity described previously, indicating that the variable essentialome is primarily composed of the unique gene repertoire of each strain. Furthermore, our analysis suggests that gene essentiality can be viewed as a continuum of fitness contribution. This was apparent for genes essential in a majority of strains (four or five). In these instances, their orthologues in the remaining strains were predominantly classified as causing a GD (Table 1). This indicates that while the absence of these genes is not strictly lethal in all genetic backgrounds, it likely imposes a significant fitness cost.

**Table 1.** Status of the remaining genes (%) not categorized ES by TNSEEK when at least one gene of the OMA orthogroup is essential

Nb of ES essential genes within the OMA orthogroup	Status of the non-ES genes* (% total)			
	GD	NE	Absent	Duplicated
6	0.0	0.0	0.0	0.0
5	76.1	13.0	8.7	2.2
4	87.5	12.5	0.0	0.0
3	47.2	37.0	13.9	1.8
2	39.7	39.7	19.9	0.6
1	10.4	26.7	87.6	0.4

* The growth advantage category is not represented in the table as none of the non-ES gene fall into this category.

In summary, the essentialome of these *Pectobacteriaceae* strains is characterized by two components: a stable, conserved core reflecting their shared biology and a larger, variable component derived from the accessory genome, which appears to shape the specific survival dependencies of each strain.

### Distinct functional signatures of the core and strain-specific essentialomes

**Fig. 4.**
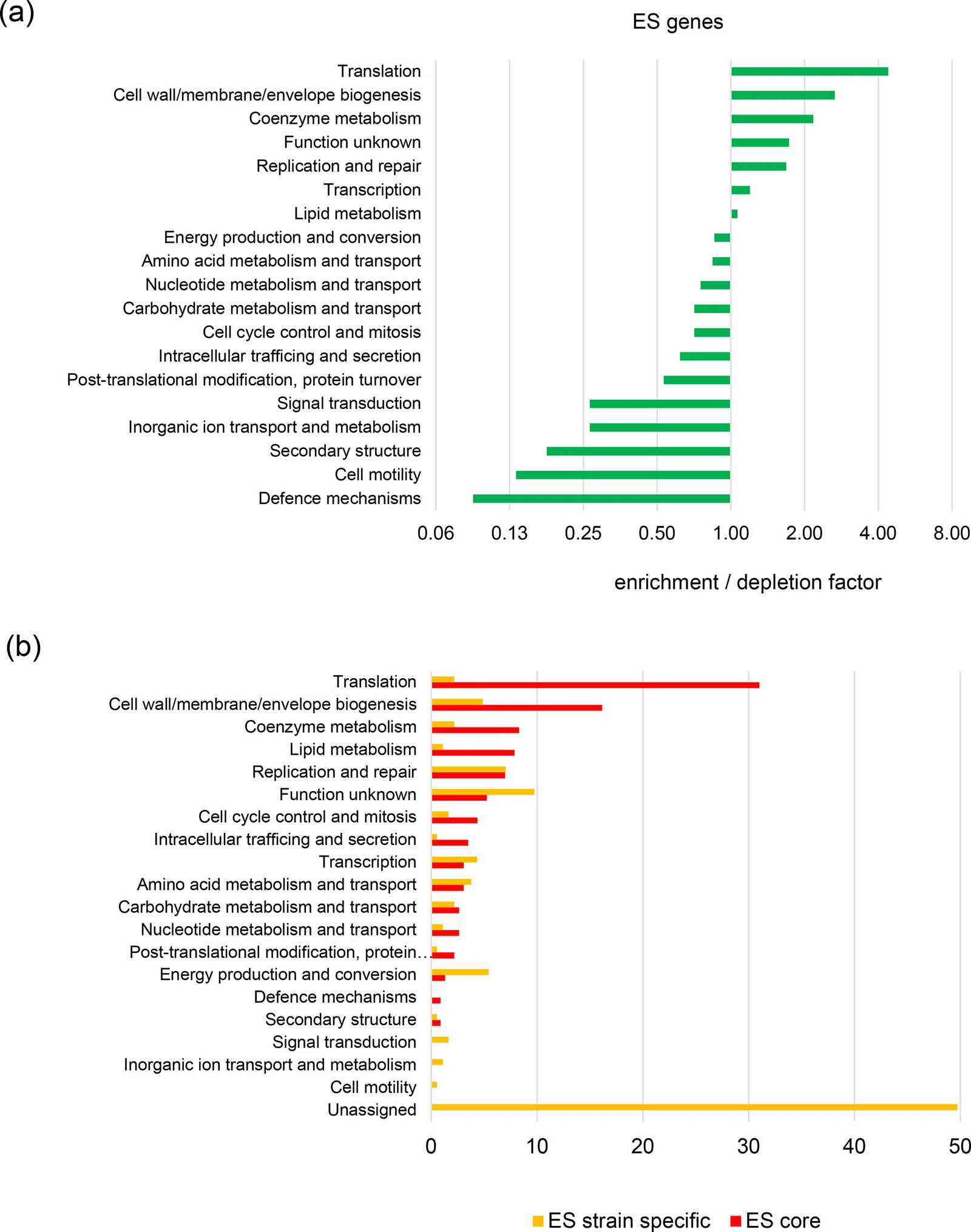
Distribution of COG categories. (a) Enrichment or depletion factor of all essential genes (ES) according to total genes for each COG category (log2 scale). Factors above 1 mean over-representation and below 1 under-representation according to the total genes. (b) Percentage of COG categories for the 181 strain-specific ES genes (yellow bars) or the 225 core ES genes (red bars). Among the strain-specific ES genes, nearly 50% are not assigned to any COG category.

To investigate the biological roles of the core and variable essentialomes, we performed a functional analysis using the Clusters of Orthologous Groups (COG) database. The results revealed clear differences in the functional profiles of these two gene sets (Fig. 4a).

First, we analysed the functional distribution of all 552 orthogroups containing at least 1 essential gene. Of these, 458 could be assigned to a COG category, while 94 remained unclassified. As expected, categories related to core cellular processes were significantly enriched. Specifically, the most overrepresented functions included ‘translation, ribosomal structure and biogenesis’ and ‘cell wall/membrane/envelope biogenesis’. In contrast, functions related to environmental interaction and adaptation, such as ‘defence mechanisms’ and ‘cell motility’, were the most underrepresented. A contrast was observed when comparing the 225 core essential genes with the 181 strain-specific essential genes (Fig. 4b). The profile of the variable essentialome was different. A high proportion (50%) of the 181 strain-specific essential genes could not be assigned to any COG category, suggesting a gap in the functional annotation of these genes. For the genes that could be classified, the most frequent designation was ‘Function unknown’, which accounted for over 10% of the set. In contrast, all 225 core essential genes have assigned COG categories, with the most prevalent categories being ‘translation’ and ‘cell wall/membrane/envelope biogenesis.’ This functional divide indicates that while the machinery for cell maintenance appears conserved, a portion of each strain’s specific dependencies may be governed by a diverse and less-characterized collection of genes.

### Comparative analysis with *E. coli* K-12 reveals the influence of growth conditions and functional redundancy

The *Pectobacteriaceae* family is close to the *Enterobacteriaceae* family within the *Enterobacterales* order [34]. To better understand the factors governing gene essentiality in *Pectobacteriaceae*, we compared the essentialome of the six SRP strains with that of the well-characterized *Enterobacterales* model, *E. coli* K-12. This comparison allowed us to identify genes with differential essentiality and to explore the biological reasons for these differences.

Our analysis identified a set of 24 genes that are considered essential for *E. coli* K-12 but were found to be NE in all 6 of the SRP strains we investigated (Table S15). A closer examination of these genes suggests that these discrepancies are not due to fundamental biological divergence but rather highlights the principle of conditional essentiality, where a gene’s requirement is dependent on the specific environmental context.

A clear illustration of this is the *cydABCDX* gene cluster. In *E. coli*, these genes encode the cytochrome bd-I quinol oxidase, a terminal oxidase in the electron transport chain. Cytochrome bd-I is assembled from three core proteins: CydA (orthogroup OMA0072), CydB (OMA01799) and CydX (QR68_06300). In addition, two accessory proteins, CydC (OMA00690) and CydD (OMA00639), function as cysteine and glutathione exporters. These transporters are essential for cytochrome maturation, as they facilitate the incorporation of haem [35]. While this complex is not required for growth at moderate temperatures, it becomes critical for survival and cell division at 37 °C, the standard culture temperature for *E. coli* [36]. Consequently, it is classified as essential under these conditions. However, our SRP strains were cultured at a lower temperature of 25 °C. At this temperature, alternative respiratory pathways are sufficient in *E. coli*, and the *cyd* genes are not essential. The NE status of these genes in our dataset is therefore consistent with the known temperature-dependent physiology of *Enterobacterales*. A similar temperature-dependent essentiality is observed for the *rpoE* gene. An *E. coli rpoE* mutant is unable to grow above 30 °C, rendering *rpoE* essential at 37 °C [37]. Importantly, *Dickeya* and *Pectobacterium rpoE* mutants exhibit no GDs at 25 °C, the temperature used in our experiments.

Furthermore, it is plausible that genes causing non-lethal GDs in *E. coli* at 37 °C may be classified as essential in our study due to the different growth conditions. For instance, genes such as *nlpD* (OMA02130), *priA* (OMA00249) and *secF* (OMA03091) are classified as NE in Goodall’s *E. coli* dataset [33]. However, mutations in these genes are known to impair growth, with the *secF* mutant, for example, displaying exacerbated defects at lower temperatures [38–40]. It is therefore possible that such GDs are more pronounced in *Dickeya* and *Pectobacterium* when cultured at 25 °C, leading to the depletion of these mutants from the population and thus an essential phenotype under our experimental conditions.

Conversely, our analysis identified 14 orthogroups essential in all 6 SRP strains but NE in *E. coli* K-12 (Table S16). The presence of paralogues and the existence of non-orthologous functional analogues in *E. coli* account for these discrepancies. For example, the six SRP strains possess only a single copy of the *gpmA* essential gene (OMA03987), which encodes a phosphoglyceromutase. In contrast, the *E. coli* genome contains in addition to *gpm*A the paralogue *gpmM* which performs the same function. Due to this duplication, *E. coli* can tolerate the loss of *gpmA*, as the function is maintained by *gpmM*. The *gpmA* gene is therefore classified as NE in this genomic context. Both genes must be disrupted simultaneously in *E. coli* to inhibit growth [41]. The second mechanism is the use of non-orthologous functional analogues, where the two organisms utilize different proteins to perform the same essential task. This is the case for the DNA helicase loader function, a critical step in the initiation of DNA replication. In *E. coli*, this essential function is encoded by the *dnaC* gene [42]. The SRP strains rely on a distinct replication-initiation factor. Proteins belonging to orthogroup OMA05632, which are essential in all six strains, are annotated as proteins of unknown function by blastp (Table S3) but contain a DciA domain detected by Interproscan [43]. DciA is not homologous to DnaC and is not itself the replicative helicase; rather, it acts as a replicative helicase operator involved in bacterial replication initiation. Thus, although the essential replication-initiation function is conserved, it is performed by evolutionarily distinct genes.

### Genus-specific patterns of gene essentiality suggest a few distinct metabolic capabilities between *Dickeya* and *Pectobacterium* genera

**Fig. 5.**
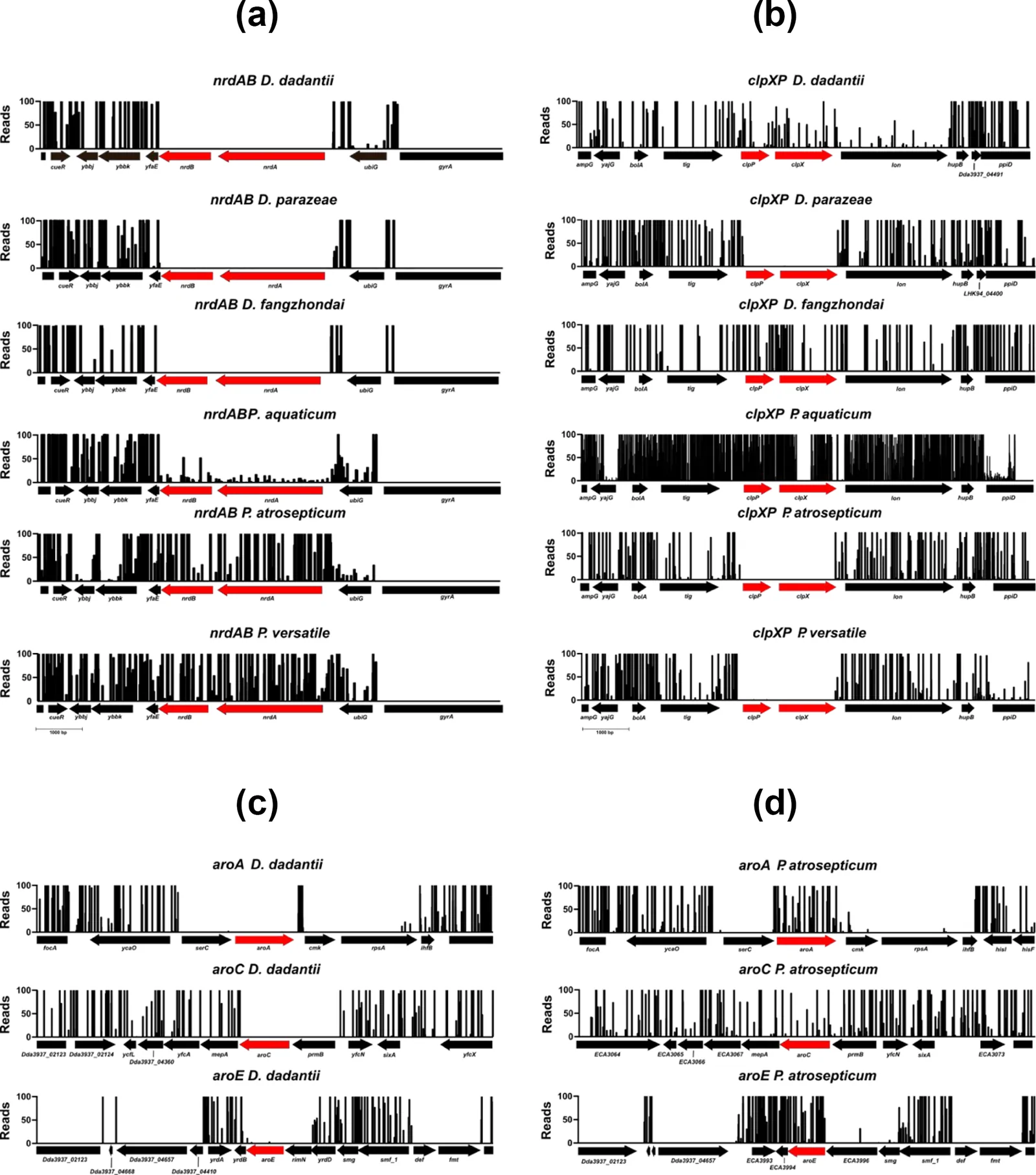
Tn-seq insertion profiles at selected loci in various *Dickeya* and *Pectobacterium* species. Each panel shows normalized Tn-seq read counts (y-axis, ‘reads’) mapped along the genomic region encompassing the indicated locus. Black peaks represent mapped insertion reads; absence of peaks indicates a lack of Tn insertions due to essentiality under the tested growth conditions. Red arrows highlight the gene(s) assessed for essentiality in each panel: (**a**) *nrdAB* cluster; (**b**) *clpXP* operon; (**c**) *aroA*, *aroC* and *aroE* in *D. dadantii*; and (**d**) *aroA*, *aroC* and *aroE* in *P. atrosepticum*. Gene organization is depicted with black arrows and gene names below each region.

Eighteen orthogroups contain genes that are essential in the three *Dickeya* species but not in the three *Pectobacterium* species (Table S13). A clear example is the d-alanine-d-alanine ligase (Ddl), which catalyses a crucial step in peptidoglycan synthesis. *Dickeya* species possess only the *ddlA* gene that codes for this activity (orthogroup OMA02306), while *Pectobacterium* species have only *ddlB* (OMA03351). Consequently, the deletion of *ddlA* is lethal in *Dickeya*, and the deletion of *ddlB* is lethal in *Pectobacterium*. In contrast, *E. coli* possesses both *ddlA* and *ddlB*, providing functional redundancy that allows it to survive the loss of either gene individually [44]. Another case involves the *nrdA* (OMA00091) and *nrdB* (OMA01483) genes, which encode the two subunits of the class Ia ribonucleotide reductase. These genes are essential in both *E. coli* and *Dickeya*, but surprisingly, they are not essential in *Pectobacterium* (Table S13) (Fig. 5a). While no duplication of *nrdA/B* was found in *Pectobacterium*, they do possess an alternative ribonucleotide reductase system, encoded by *nrdE* and *nrdF*. This alternative enzyme, which is absent in *Dickeya*, is known to function in *E. coli* under specific conditions, such as iron-depleted, manganese-rich conditions [45]. Further investigation is needed to confirm if the *nrdE/F* system provides a functional substitute for *nrdA/B* in *Pectobacterium* under the growth conditions used in this study.

Conversely, a more limited set of genes was identified as essential specifically within the *Pectobacterium* genus. Only three orthogroups contain genes that are essential in the three studied *Pectobacterium* species but not in the three *Dickeya* species (Table S14). OMA04333 includes essential orthologous genes in each *Pectobacterium* species (DMB82_0011620, ECA2435 and LLE50_21950). Based on sequence similarity and domain architecture, these proteins are orthologues of RdgA, a regulator previously identified in *P. versatile* strain 71. RdgA is linked to the control of pectin lyase production and, by homology with *E. coli*, to DNA repair and stress-response pathways [46]. We hypothesize that these RdgA orthologues act as stress-responsive repressors to coordinate DNA damage repair, control expression of potentially deleterious genes during rapid growth in rich media and thereby maintain cellular homeostasis and viability. Given their essentiality in *Pectobacterium*, experimental characterization of their precise role is warranted.

Distinct essentiality patterns of shikimate pathway genes were also observed between *Dickeya* and *Pectobacterium* when grown in TSB 50%. Specifically, in *Dickeya,* the genes *aroA*, *aroC* and *aroE* are essential, while these same genes are NE in *Pectobacterium* (Fig. 5c, d). These genes encode critical enzymes responsible for aromatic amino acid biosynthesis, culminating in the production of chorismate that is vital for synthesizing phenylalanine, tyrosine, tryptophan and other essential metabolites. Additional observations show that *aroF*, encoding a tyrosine-sensitive DAHP synthase catalysing the first committed step, exhibits a GD phenotype in *Dickeya* but is NE in *Pectobacterium*, while *aroQ*, encoding a type II 3-dehydroquinate dehydratase, remains essential in both genera. Two other DAHP synthase isoenzyme genes, *aroG* and *aroH*, as well as the aromatic amino acid permease gene *aroP*, are NE in both. This pattern suggests that *Dickeya* more heavily depends on *de novo* aromatic amino acid biosynthesis for growth, even in nutrient-rich conditions, whereas *Pectobacterium* compensates for pathway disruption effectively, likely through superior transport and utilization of environmental amino acids and peptides. Proposed hypotheses to explain these genus-specific differences include greater abundance or affinity of aromatic amino acid transporters and enhanced peptide uptake and hydrolysis systems in *Pectobacterium*, allowing it to scavenge sufficient aromatic amino acids from the complex peptides in TSB medium. Additionally, evolutionary divergence in metabolic regulation and differences in the demand for chorismate-derived compounds may contribute to these distinct physiological strategies.

### Defence-related systems reveal strain-specific essential restriction–modification, toxin–antitoxin and contact-dependent growth inhibition systems

Defence-related systems are crucial for protecting bacterial cells from foreign genetic elements like bacteriophages and plasmids. To systematically identify the essential genes carried by these modules, we analysed all six genomes using the DefenseFinder tool. This analysis revealed that the vast majority of defence genes are NE for growth in rich medium. Specifically, out of 197 identified genes belonging to anti-phage systems, 152 were categorized as NE. Nevertheless, by comparing the predicted defence systems (Table S17) with our catalogue of essential genes, we identified a critical subset of 14 essential anti-phage genes belonging to 8 distinct system types. This finding suggests that cognate toxic, restriction or immunity functions are constitutively active under our experimental conditions, thus making the corresponding protective component essential even in the absence of an exogenous phage challenge.

Several of these essential genes are part of active restriction–modification (R–M) systems. In these systems, a methyltransferase enzyme modifies specific DNA sequences in the host genome, protecting it from cleavage by a corresponding restriction endonuclease. This mechanism becomes essential for cell survival if the restriction enzyme is constitutively expressed. A clear example is found in *D. fangzhongdai*, where the gene QR68_02190, encoding a DNA cytosine methyltransferase, was identified as essential (Table S17). This gene is located adjacent to QR68_02195, which encodes the restriction enzyme MvaI which recognizes the sequence 5′-CCAGG-3′ and is inhibited by the methylation of the internal cytosine residue [47, 48]. The essentiality of the methyltransferase (QR68_02190) implies that the MvaI restriction enzyme is actively expressed in the TSB 50% medium, making protective methylation a prerequisite for survival. A similar configuration was observed in *D. parazeae*, where the essential gene LHK94_01575 encodes a site-specific DNA methyltransferase (Table S17). This enzyme is homologous to the SmaI methyltransferase from *Serratia marcescens*, which targets the palindromic sequence 5′-CCCGGG-3′. Located in its genomic vicinity is LHK94_01580, which encodes a type II restriction enzyme homologous to the SmaI enzyme. Therefore, the essentiality of the methyltransferase gene LHK94_01575 is required to prevent the LHK94_01580 enzyme from cleaving the chromosome. These two examples show that specific type II R–M systems are essential in *D. fangzhongdai* and *D. parazeae*. This contrasts with many other methyltransferases identified by DefenseFinder, which were classified as NE in our analysis. For these NE systems, it is likely that their cognate restriction enzymes are either absent from the genome or not actively expressed under the tested conditions.

The analysis of defence systems revealed that several toxin–antitoxin (TA) systems exhibit diverse essentiality profiles across the studied strains (Table S17). These systems typically function as addiction modules to ensure their stable inheritance. The essentiality of an antitoxin gene is a strong indicator that its corresponding toxin is actively expressed, making the antitoxin’s neutralizing function critical for host viability. DefenseFinder also identified several TA systems with diverse essentiality profiles. Among these, the RosmerTA system consists of the RmrA antitoxin, a Zn-dependent peptidase and the RmrT toxin, which induces membrane depolarization [49]. While the RmrT toxin shows sequence variability, the TA system is conserved in *D. parazeae* (LHK94_20535/LHK94_20530), *P. atrosepticum* (ECA2912/ECA2211) and *P. versatile* (LLE50_14920/LLE50_14925). In these three species, the *rmrA* antitoxin gene was essential, strongly suggesting that the RmrT toxin is active under our experimental conditions. Intriguingly, RmrT toxins have previously been shown to be responsible for the essentiality of the ClpX chaperone in *S. pneumoniae* [50] and the ClpP protease in *E. coli* E101 [51]. Our results reveal a compelling correlation that supports this model: the genes encoding the ClpX protease (OMA01412) are essential exclusively in the three strains (*D. parazeae*, *P. atrosepticum* and *P. versatile*) that contain the RosmerTA system (Fig. 5b).

Another essential module identified is the AbiE system, a type IV TA system [52] present in *P. aquaticum* and *P. versatile* (Table S17). This system is encoded by a bicistronic operon that includes the toxin AbiEii, a guanosine triphosphate-binding nucleotidyltransferase that causes reversible growth arrest, and the antitoxin AbiEi, which acts as a transcriptional repressor by binding the *abiE* promoter. In both *P. aquaticum* and *P. versatile*, the antitoxin genes (DMB82_0015475 and LEE50_14595, respectively; AbiEi_3-orthogroup OMA07585) were found to be essential, indicating active expression of the corresponding toxin genes (DMB82_0015470 and LLE50_14590; AbiEii-orthogroup OMA06941). In contrast, *D. parazeae* possesses a homologous antitoxin gene (LHK94_08840) that is NE. This is explained by the absence of the cognate toxin gene of the orthogroup OMA06941 in its genome (Table S3).

A final example found by DefenseFinder involves a type II TA system belonging to the RelE/ParE family. In *P. aquaticum*, the gene (DMB82_0003135), which encodes an antitoxin characterized by a C-terminal XRE-HTH domain, was identified as essential (Table S17). This antitoxin is associated with its cognate toxin encoded by the adjacent gene (DMB82_0003140). The essentiality of this antitoxin is conserved in related species possessing homologous TA systems, such as *P. atrosepticum* (ECA0673/ECA0674) and *D. fangzhongdai* (QR68_07805/QR68_07810). A notable exception is observed in *P. versatile*, where the antitoxin gene (LLE50_14865) is NE despite the presence of its cognate toxin gene (LLE50_14870), suggesting a different regulatory mechanism or lack of toxin expression under the studied conditions.

This principle of an immunity protein being essential to neutralize a cognate toxin extends beyond canonical TA systems to those involved in intercellular competition. In the *D. dadantii* 3937 genome, three ES genes, *Dda3937_02097*, *Dda3937_01757* and *Dda3937_04645*, unique to *D. dadantii* 3937, encode immunity proteins that protect the producing strain from self-intoxication by contact-dependent growth inhibition (CDI) systems (Table S7). *Dda3937_02097*, also known as *cdiI*, encodes an immunity protein that neutralizes the CdiA toxin delivered via a classical CDI system [53]. Similarly, *Dda3937_01757* (*rhsIA*) and *Dda3937_04645* (*rhsIB*) encode immunity proteins specific to RhsA and RhsB toxins, respectively [54]. The essential function of the three genes strongly indicates that their cognate toxins are actively expressed under the tested conditions and exert a toxic effect in the absence of their immunity partners.

### Essential genes within prophage regions suggest lysogeny and defensive roles

**Fig. 6.**
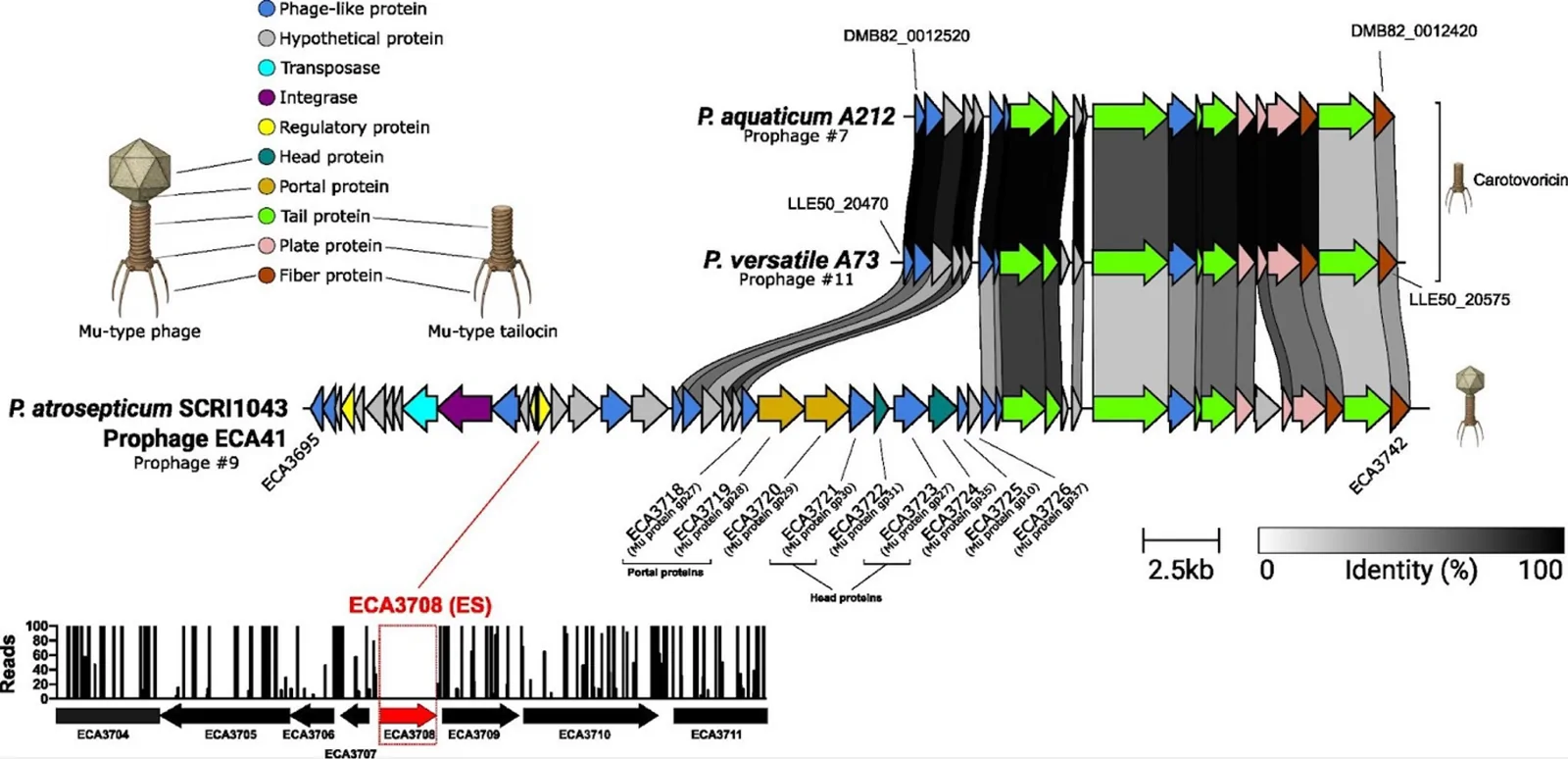
Comparative genomic analysis of a functional Mu-like prophage and two defective tailocin-encoding prophages in *Pectobacterium* species. Genomic alignment, generated using clinker, of prophage regions from three *Pectobacterium* strains. The bottom line displays the Mu-like prophage #9 (ECA41) from *P. atrosepticum* SCRI1043. This prophage contains the essential (ES) regulatory gene *ECA3708*, highlighted in red, which is required to maintain lysogeny. Genes encoding structural proteins, such as portal and head components, are also annotated. In contrast, the middle and top lines show prophage #11 from *P. versatile* A73 and prophage #7 from *P. aquaticum* A212. These regions are defective prophages that constitute the carotovoricin operons, which encode a Mu-type tailocin, a potent antibacterial weapon. Arrows represent predicted genes, coloured by their putative function according to the key. Grey links between the regions illustrate amino acid identity, with the colour intensity corresponding to the percentage of identity. The bottom histogram shows the distribution of Tn-seq reads mapped across the prophage region in *P. atrosepticum*, reflecting the transposon insertion profile. The absence of insertions (i.e. no reads) in the essential gene ECA3708 (highlighted in red) indicates its requirement for bacterial viability under the tested conditions, while neighbouring genes with higher insertion counts are dispensable.

To assess the presence of potentially essential genes within prophage regions, we employed the PHASTEST tool [55] to analyse the six SRP genomes. PHASTEST identified a total of 13 intact prophage regions across the six genomes: 1 in *D. fangzhongdai*, 2 each in *D. dadantii* 3937, *D. parazeae* A586, *P. aquaticum* A212 and *P. atrosepticum* SCRI1043 and 4 in *P. versatile* A73 (Tables 2 and S18). Prophages #2, #3, #7 and #11 are defective prophages encoding tailocins, bacterial phage tails active as antibacterial weapons conferring a significant competitive advantage within the plant niche [56] (Fig. 6). Prophages #8 and #9 of *P. atrosepticum* have been studied by Evans *et al*. [57] and are named ECA29 and ECA41, respectively. The number of essential genes per prophage varied from 0 to 4, with 11 out of the 13 prophages harbouring at least 1 essential gene (Tables 2 and S18).

**Table 2.** Phage essential genes

Strain	Prophage no./name	Region length (kb)	Region position	First locus tag*	Last locus tag*	No. of ES genes	Putative phage repressor		List of ES genes
							Description	Locus tag	
*D. dadantii* 3937	1	38	915372 – 952946	Dda3937_01762	Dda3937_02545	4	C1	Dda3937_01765	Dda3937_01763 Dda3937_01765 Dda3937_02545 Dda3937_02546
	2 dickeyocin†	28	2733074–2760902	Dda3937_03777	Dda3937_00104	1	C1	Dda3937_03777	Dda3937_03777
*D. parazeae* A586	3 dickeyocin†	25	1993353–2018459	LHK94_18375	LHK94_18525	1	C1	LHK94_18525	LHK94_18525
	4	59	2446385–2505189	LHK94_20390	LHK94_20710	2	XRE-family HTH transcriptional regulator, peptidase s24 domains	LHK94_20615	LHK94_20495 LHK94_20530
*D. fangzhongdai* B16	5	28	2806765–2834379	QR68_12205	QR68_12360	1	C1	QR68_12205	
*P. aquaticum* A212	6	55	662735–718114	DMB82_0003125	DMB82_0003450	3	C1	DMB82_0003205	DMB82_0003135 DMB82_0003185 DMB82_0003205
	7 carotovoricin†	20	2653251–2673677	DMB82_0012420	DMB82_0012520	0			
*P. atrosepticum* SCRI1043	8 ECA29 prophage‡	31	2 935 264–2 966 783	ECA2598	ECA2637	1	C1	ECA2636	ECA2636
	9 ECA41 prophage‡	36	4 144 591–4 180 799	ECA3695	ECA3742	1	Helix–turn–helix-XRE-transcriptional regulator	ECA3708	ECA3708
*P. versatile* A73	10	36	217090–252597	LLE50_12330	LLE50_12560	2	C1	LLE50_12345	LLE50_12345 LLE50_12555
	11 carotovoricin†	22	2022299–2043834	LLE50_20470	LLE50_20575	0			
	12	47	3215138–3261670	LLE50_03075	LLE50_03375	1	C1	LLE50_03110	LLE50_03110
	13	37	3595501–3632713	LLE50_04915	LLE50_05140	2	Helix–turn–helix-XRE-transcriptional regulator	LLE50_05055	LLE50_05050 LLE50_05055

* For *D. dadantii* 3937, the locus tags are not ordered.

† [57].

‡[65].

The most frequently identified essential gene function was prophage repression. Specifically, essential CI-type repressors were found in eight prophage regions. CI repressors maintain the prophage in its lysogenic state by preventing the expression of genes required for the lytic cycle. Loss of CI function would typically trigger prophage excision and cell death via lysis. For instance, the essential classification of *D. dadantii* 3937 gene *Dda3937_01765*, which encodes a CI repressor, directly reflects its role in preventing the lytic cascade. Furthermore, three other prophage regions harbour an essential gene encoding a helix–turn–helix (HTH) XRE transcriptional regulator. XRE-type regulators are a broad family of bacterial proteins known to modulate gene expression via HTH DNA-binding domains and are often involved in various processes, including phage–host interactions. These XRE proteins likely serve a function analogous to the CI repressor (Table 2) [58, 59]. Collectively, these findings suggest that 11 of the 13 identified prophage regions likely encode functional lysogenic phages and dickeyocins. Beyond the repressors, other essential genes were identified in the prophage regions (Table 2). For instance, the *Dda3937_01763* gene (prophage #1) encodes an ASCH domain-containing protein with endoribonuclease activity, potentially acting to prevent virion production. Additionally, a functional TA pair was identified in prophage #10 of *P. versatile* (Table S17), where the encoded antitoxin (*LLE50_12555*) is essential.

## Discussion

Tn-seq is a powerful tool to study bacterial strain fitness in various environments. However, to disentangle strain-specific essential fitness traits from those affecting more broadly the studied species, genus, or family, it is imperative to study and compare several strains. For this purpose, we created the TNSEEK pipeline that allowed a straightforward and easy comparison of all the Tn-seq data. We used TNSEEK to compare the results of Tn-seq experiments performed on TSB 50% for six strains belonging to two genera within the *Pectobacteriaceae* family.

The core essentialome of the *Pectobacteriaceae*, which comprises 225 essential genes conserved in the six studied bacteria, has almost the same size as those defined for the *Enterobacteriaceae* family (201 genes/13 strains from 5 genera) [7]. Interestingly, the core essentialome defined for the single species *S. pneumoniae* (206 genes/36 strains studied) is also of a similar size [8]. This suggests a lack of connection between essentiality and phylogeny, as was previously observed in another study [51]. Our rarefaction analysis showed that the six-strain dataset mostly captures the conserved essentialome, suggesting that analysing six strains is sufficient to identify the core set of essential genes at the family level, as has been observed for *P. aeruginosa* at the species level [60].

Our results are broadly consistent with recent comparative Tn-seq studies showing a conserved core essentialome together with rapid turnover of accessory essentiality. In *Enterobacteriaceae*, Ghomi *et al*. identified a limited family-level core essentialome and substantial strain-level variation in essential gene content [7]. Similarly, Rosconi *et al*. showed in *S. pneumoniae* that the pan-genome contributes extensively to strain-dependent essentiality [8].

Our work also confirmed that, following Tn-seq analysis, essentiality can be viewed as a fitness continuum. Indeed, the TNSEEK pipeline revealed that most genes that were essential in four to five of the analysed strains were categorized as GDs in the other tested strains. This is due to the fact that, in a Tn-seq screen, each mutant competes with the population of bacterial cells that are functional for the same character. Therefore, most mutations that lead to a loss of fitness will either decrease in frequency or be eliminated from the population, resulting in GD or ES categorization, respectively.

Comparing our Tn-seq analysis with one performed on *E. coli* K12 provided potential explanations for some of the differences between the two studies. Firstly, while a previous study suggested that a shift in essentiality is not associated with a shift in expression level [51], we found that the choice of growth conditions, particularly temperature, can significantly influence the determination of the essential gene set and account for some of the observed results. Secondly, duplication events occurring in one strain but not the other could also influence the outcome of Tn-seq experiments. In-depth analysis also supports this latter explanation for some of the differences in essentiality observed between the six strains studied. Additionally, alternative enzymes that fulfil the same essential function, or differential gene regulation between strains, were also identified as potential explanations for the differences between the two studies.

Our comparative analysis also identified a large set of 181 species-specific essential genes, ranging from 39 in the case of *P. atrosepticum* to 16 in the case of *P. versatile*. Most of these species-specific ES genes are absent from the genomes of the other five analysed species, which highlights the importance of variable genomes for essentiality. As only one strain was studied per species, it was not possible to determine whether the observed specificity is linked to the analysed species or the specific strain studied. Future Tn-seq analyses involving a set of strains from the same species could distinguish between ES-specific genes that are conserved at the species level and those that are strain-specific. Interestingly, COG analysis did not assign most of these ES-specific genes to any known function. This contrasts with core essential genes, which are mostly associated with the maintenance of bacterial cell functions. The essentiality of a large number of genes with unknown functions highlights the importance of conducting functional studies in future.

Our focused analysis of the defensome indicated that a few of the ES gene functions were related to various defence mechanisms including R–M systems, TA mechanisms and CRISPR proteins for example. The essentiality of selected components of defence-associated loci in TSB 50%, in the absence of a known stressor or bacterial competition, suggests that a few cognate restriction, toxin or immunity activities impose a constitutive dependency. This is consistent with the genetic organization of R–M systems, TA systems and CDI/Rhs immunity modules, but it will require targeted experimental validation. Notably, the mechanisms responsible for this constitutive frontline defence vary greatly among the tested strains, highlighting its high evolvability. These species-specific patterns suggest that distinct regulatory architectures, ecological pressures or metabolic trade-offs determine which defence elements are constitutively active and indispensable. Deepening the investigation into these context-dependent essentialities may uncover the evolutionary forces shaping bacterial defensome repertoires. These species-specific patterns within defence gene deployment are also consistent with experimental work performed on SRP which demonstrated that competition mechanisms are complex and mainly occur between strains, regardless of the species concerned [61].

Phage repressors were found to be essential for most of the phage regions identified by PHASTEST, regardless of whether the region encoded a putative functional phage or a phage remnant such as the tailocin dickeyocin of the *Dickeya* genus [56]. Similar essentiality of phage repressor in rich medium was previously reported in dense transposon insertion libraries of Salmonella serovars Typhi and Typhimurium [62]. This indicates that these phage regions require repression in TSB 50% in the absence of a known inducing stressor. Such tight repression is probably necessary to maintain lysogeny and prevent deleterious prophage induction. Dickeyocin production is induced by various stresses such as mitomycin C, reactive oxygen species and antibiotics [56, 63]. By contrast, previous research on the two complete prophages of *P. atrosepticum* SCRI1043, the Mu-like ECA41 (prophage #9) and the P2-like ECA29 (prophage #8), demonstrated their resistance to chemical or physical induction [64], suggesting that an unknown induction mechanism required to induce their lytic cycle remains to be discovered. Our work also revealed a fundamental regulatory distinction between the *Dickeya* tailocins dickeyocin and the *Pectobacterium* tailocin carotovoricin [65]. Carotovoricin-producing elements lack a dedicated essential repressor gene directly associated with the carotovoricin clusters (Table 2). This absence suggests that their activation mechanism diverges from the standard transcriptional repression model controlling both complete prophages and dickeyocins. Instead, carotovoricin synthesis is known to be regulated by the bacterial SOS response via the global repressor LexA [66]. The essential nature of the *lexA* gene across all *Pectobacterium* strains analysed (OMA04984) aligns with its vital role in this distinct regulatory pathway. In addition to phage repression, the presence of a functional TA pair was revealed within one of the phage regions through the essentiality of the antitoxin gene. As with many moron genes carried within phages, this T pair likely contributes to bacterial strain fitness [63].

In conclusion, the TNSEEK platform, which was specifically developed for this project to compare the results of Tn-seq experiments performed on bacteria from several species and genera, has proven its proficiency here.

Application to Tn5-based or non-TA-specific transposon libraries would require adaptation of the preprocessing and insertion-site definition steps.

## Supplementary material

10.1099/mgen.0.001762 Supplementary Material 1.

## Abbreviations

CDI: contact-dependent growth inhibition
COG: Clusters of Orthologous Groups
ES: essential
GD: growth defect
HTH: helix–turn–helix
LB: Luria–Bertani
NE: non-essential
OD_600_.U: OD units at 600 nm
OMA: Orthologous Matrix
R–M: restriction–modification
SRP: soft rot *Pectobacteriaceae*
TA: toxin–antitoxin
Tn-seq: transposon sequencing
TSB: tryptic soy broth
TTR: trimmed total reads
